# Tumor Size Is a Critical Factor in Adjuvant Chemotherapy for T_3-4a_N0M0 Gastric Cancer Patients after D2 Gastrectomy

**DOI:** 10.1155/2017/4928736

**Published:** 2017-02-26

**Authors:** Shi Chen, Li-Ying Ou-Yang, Run-Cong Nie, Yuan-Fang Li, Jun Xiang, Zhi-Wei Zhou, Ying-Bo Chen, Jun-Sheng Peng

**Affiliations:** ^1^The 6th Affiliated Hospital, Sun Yat-sen University, No. 26, Yuancun Erheng Road, Tianhe District, 510655 Guangzhou, China; ^2^Department of Intensive Care Unit, Sun Yat-sen University Cancer Center, 651 Dongfeng East Road, 510060 Guangzhou, China; ^3^Department of Gastropancreatic Surgery, Sun Yat-sen University Cancer Center, 651 Dongfeng East Road, 510060 Guangzhou, China

## Abstract

*Aim*. To investigate whether tumor size is a reasonable indication for adjuvant chemotherapy for T_3-4a_N0M0 gastric cancer patients after D2 gastrectomy. *Method*. We performed a retrospective study of 269 patients with a histological diagnosis of T_3-4a_N0M0 stage gastric cancer who underwent D2 radical surgery at the Sun Yat-sen University Cancer Center or the Sixth Affiliated Hospital of Sun Yat-sen University between January 2006 and December 2010. The follow-up lasted until June of 2015. Chi-square tests and Kaplan-Meier methods were employed to compare the clinicopathological variables and prognoses. *Result*. For this group of patients, univariate analyses revealed that tumor size (*p* < 0.001), pathological T stage (*p* < 0.001), and tumor location (*p* = 0.025) were significant prognostic factors. Adjuvant chemotherapy did not exhibit prognostic benefits. For patients with tumors larger than 5 cm, univariate analysis revealed that tumor location (*p* = 0.007), Borrmann type (*p* = 0.039), postoperative chemotherapy (*p* = 0.003), and pathological T stage (*p* < 0.001) were significant prognostic factors. Multivariate analysis revealed that postoperative chemotherapy and pathological T stage were independent prognostic factors. *Conclusion*. Our results imply that tumor size should be a critical factor in the decision to utilize adjuvant chemotherapy for T_3-4a_N0M0 gastric cancer patients after D2 gastrectomy. Additional randomized controlled trials are required before this conclusion can be considered definitive.

## 1. Background

Gastric cancers are the fourth most common malignancies worldwide, and they are the second most lethal [1–3]. Gastrectomy with D2 lymphadenectomy is recommended as a standard surgery for gastric cancer patients and results in improved overall survival [4–6]. Moreover, adjuvant chemotherapy has been proven to improve the overall survival of advanced gastric cancer patients after D2 gastrectomy [7, 8]. However, for N0 patients, particularly T3 and T4a patients, the use of adjuvant chemotherapy remains controversial. Although N0-group patients were not found to benefit from adjuvant chemotherapy in an ACTS trial, stage II gastric cancer patients without lymph node metastases were not separately analyzed, and there were only 112 patients in the N0 group [7]. Moreover, in the CLASSIC trial, the N0 group also exhibited no survival benefit following adjuvant chemotherapy [8]. Thus, the question of how to select N0 patients for adjuvant chemotherapy, particularly stage II patients, remains unresolved. The role of postoperative chemotherapy in T3-T4a gastric cancer patients is still controversial. In addition to TNM stage, other risk factors should be identified for this patient group to select for whom postoperative chemotherapy would be beneficial. Tumor size is also an important characteristic of gastric cancer, and we found that it was an informative factor for chemotherapy selection.

Tumor size is another factor that can be evaluated in gastric cancer patients, although it is not listed in the staging systems of the UICC or JGCA for gastric cancer [9, 10]. Obviously, larger tumors are more advanced. In the present study, we performed a retrospective analysis that focused on these N0-group gastric cancer patients, compared the prognoses according to different tumor size groups, and attempted to determine the prognostic value of tumor size in relation to adjuvant chemotherapy.

## 2. Materials and Methods

### 2.1. Ethics Statement

All of the patients provided written informed consent for their information to be stored in a hospital database. We obtained separate consent for the use of this information for research. Study approval was obtained from independent ethics committees at the Sixth Affiliated Hospital of Sun Yat-sen University and the Cancer Center of Sun Yat-sen University. This study was undertaken in accordance with the ethical standards of the World Medical Association Declaration of Helsinki.

### 2.2. Patient Inclusion and Exclusion Criteria

The inclusion criteria were as follows: (1) WHO performance status of 0 to 1; (2) histologically proven T3-4 adenocarcinoma of the stomach without evidence of lymph node metastasis; (3) no prior gastric surgery; (4) no previous radiotherapy or other treatments, including immunotherapy or traditional Chinese medicine; and (5) no synchronous or metachronous cancers.

### 2.3. Chemotherapy

Various chemotherapeutic regimens were considered in our research: 36 patients received Xeloda (1000 mg/m^2^, D1–14, Q3W, cycles: 5.67 ± 1.15); 67 patients received the XELOX regimen (oxaliplatin: 130 mg/m^2^ D1 + Xeloda 1000 mg/m^2^, D1–14, Q3W, cycles: 5.53 ± 1.55); and 44 patients received the FOLFOX regimen (oxaliplatin: 85 mg/m^2^ D1 + CF 400 mg/m^2^ D1 + 5-Fu 2800 mg/m^2^, D1-D2, Q2W, cycles: 8.52 ± 1.57). Of another 33 patients, 14 received the S-1 regimen (40–60 mg, bid, D1–14, Q3W, cycles: 5.71 ± 1.43); 13 received the CX regimen, (cisplatin: 60 mg/m^2^ D1 + Xeloda 1000 mg/m^2^, D1–14, Q3W, cycles: 4.92 ± 1.50); 5 received the SOX regimen (oxaliplatin: 85 mg/m^2^ D1 + S-1 1000 mg/m^2^, 40–60 mg, bid, D1–14, Q3W, cycles: 4.92 ± 1.50); and one received the DX regimen (docetaxel: 75 mg/m^2^ D1 + Xeloda 1000 mg/m^2^, D1–14, Q3W, cycles: 5).

### 2.4. Patient Characteristics

**Figure 1:**
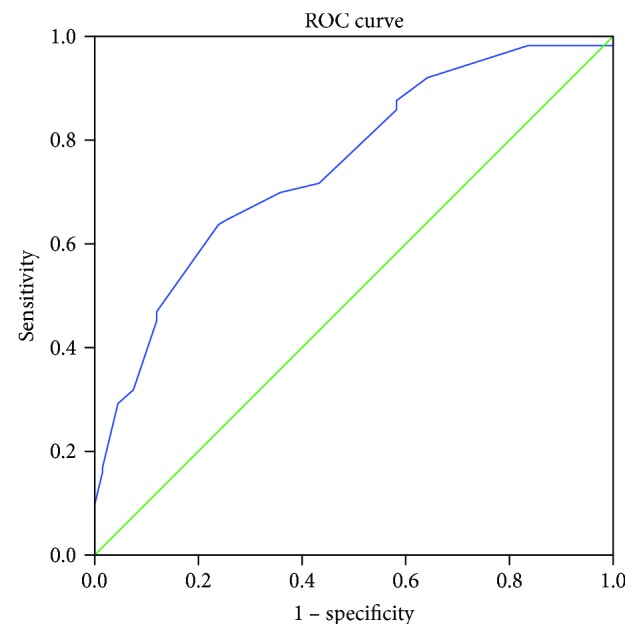
The AUC was 0.751, and the largest Youden index was 0.398, corresponding to a tumor size of 4.75 cm. However, we believed that, in the clinic, 5 cm is a more appropriate cut-off value for doctors seeking to decide whether the patient should receive postoperative chemotherapy.

From January 2006 to December 2010, 269 consecutive patients with a histological diagnosis of T3-4N0 gastric cancer who underwent D2 radical surgery at the Sixth Affiliated Hospital of Sun Yat-sen University or the Sun Yat-sen University Cancer Center were included in this study. We divided the patients according to tumor size. We analyzed the ROC curve data and considered two balanced arms, selecting 5 cm as the cutoff value (Figure 1). Patients with gastric tumors of less than 5 cm were included in the small gastric cancer group, and patients with tumors greater than 5 cm were included in the large gastric cancer group. The clinicopathological factors are presented in Table 1.

### 2.5. Follow-Up

After treatment, the patients were monitored every month for the first year, every 3 months for the second year, and every 6 months thereafter, with regular follow-up assessments. Telephone calls and letters were used to follow up on the patients who were not able to attend regular follow-up assessments. Complete data were collected for all 269 patients through December 2014. The following-up period ranged from 6 months to 90 months (median: 46 months).

### 2.6. Statistical Methods

A chi-square test was used to compare the categorical variables between the palliative operation group and the other groups. Student's *t*-tests were used to compare the continuous variables. Univariate survival analyses were performed using Kaplan-Meier methods. The survival curves were compared with the log-rank test. The statistical analyses were performed with SPSS software version 20.0 (SPSS Inc., Chicago, IL) for Windows. Statistical significance was defined as *p* < 0.05.

## 3. Result

### 3.1. Univariate Analyses of the Prognoses of Gastric Cancer Patients

**Figure 2:**
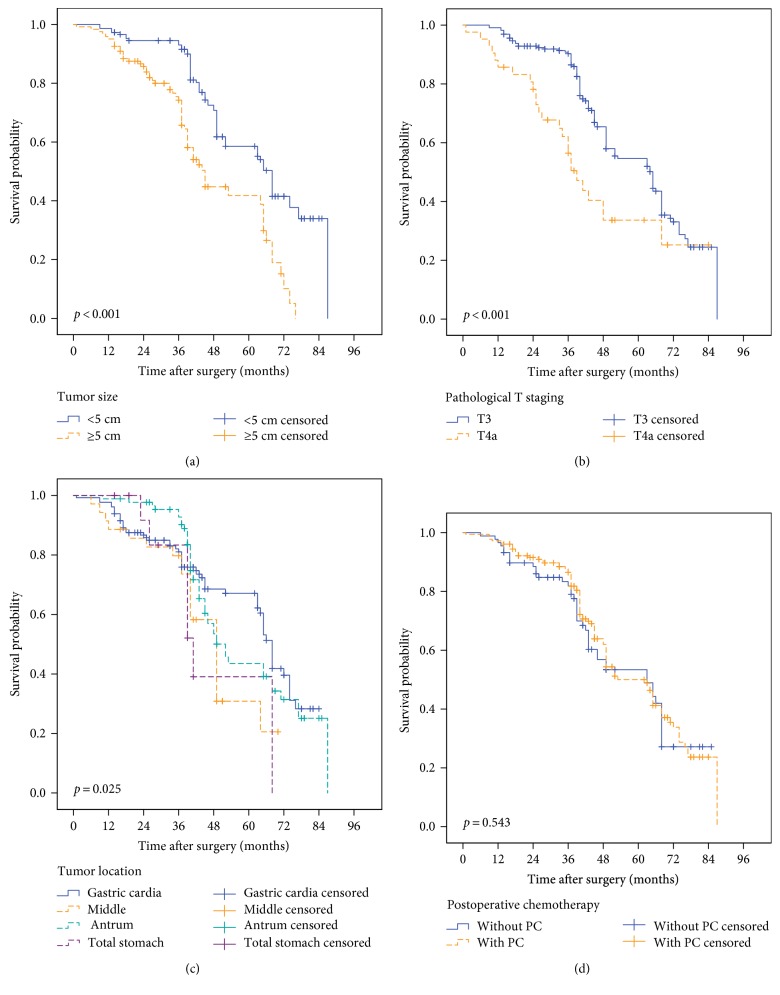
Univariate analysis of 267 T_3-4a_N0M0 gastric cancer patients. (a) The mean survival times of patients with tumor sizes smaller than 5 cm and larger than 5 cm were 63.25 and 47.95 months, respectively (*p* < 0.001). (b) The mean survival times of the T3 and T4a patients in the study were 59.61 and 45.89 months, respectively (*p* < 0.001). (c) Tumor location was also a prognostic factor for this group of patients (*p* = 0.025). (d) Adjuvant chemotherapy did not have a prognostic benefit for this group of gastric cancer patients (*p* = 0.543).

According to the Kaplan-Meier analysis, tumor size (*p* < 0.001), pathological T stage (*p* < 0.001), and tumor location (*p* = 0.025) were risk factors (as shown in Table 2). However, no significant survival difference was found between the patients with postoperative chemotherapy and those without postoperative chemotherapy. The median survival times of the patients who received and did not receive postoperative chemotherapy were 58.0 months and 56.1 months, respectively (*p* = 0.543). The survival curves are illustrated in Figure 2.

### 3.2. Multivariate Analysis of the Prognoses of Gastric Cancer Patients

Furthermore, we used the Cox regression model to analyze these risk factors in order to identify the independent risk factors. The results revealed that tumor size, tumor location, and pathological T stage were the only independent prognostic risk factors. All of these results are presented in Table 3.

### 3.3. Postoperative Chemotherapy Brings No Benefits for Stage II Gastric Cancer Patients with Tumors Less Than 5 cm in Size

**Figure 3:**
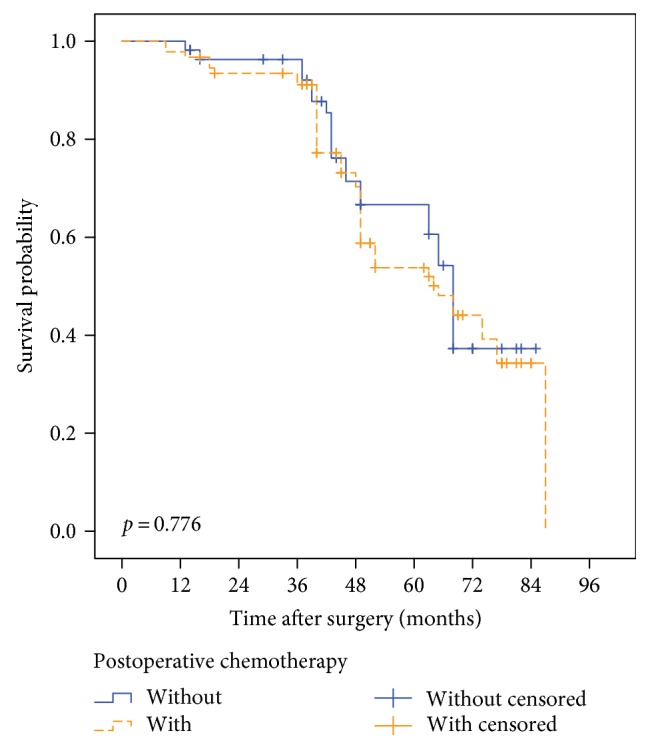
In the group of patients with tumor sizes of less than 5 cm, the median survival times of the chemotherapy and without chemotherapy groups were 64.43 months and 62.38 months, respectively (*p* = 0.776).

In the group of patients with tumor sizes of less than 5 cm, the postoperative chemotherapy did not show any benefit. As shown in Figure 3, the median survival times of the chemotherapy and without chemotherapy groups were 64.43 months and 62.38 months, respectively (*p* = 0.776).

### 3.4. Univariate Analyses of the Prognoses of Gastric Cancer Patients with Tumors Greater Than 5 cm in Size

**Figure 4:**
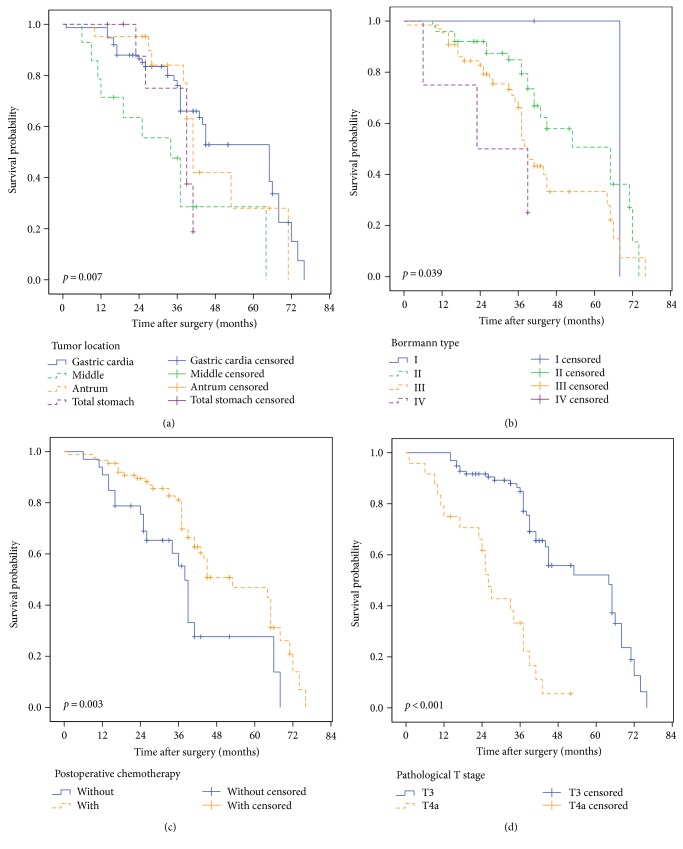
Univariate analysis of the prognosis of gastric cancer patients with tumor sizes larger than 5 cm. (a) The tumor location (*p* = 0.007), (b) Borrmann type (*p* = 0.039), (c) postoperative chemotherapy (*p* = 0.003), and (d) pathological T staging (*p* < 0.001) were the prognostic factors for these gastric cancer patients.

We first compared the clinicopathological factors between the postoperative chemotherapy and no postoperative chemotherapy groups of gastric cancer patients with tumors greater than 5 cm (Table 4). Kaplan-Meier analysis revealed that tumor location (*p* = 0.007), Borrmann type (*p* = 0.039), postoperative chemotherapy (*p* = 0.003), and pathological T stage (*p* < 0.001) were prognostic risk factors (Table 5). The survival curves are illustrated in Figure 4.

### 3.5. Multivariate Analysis of the Prognoses of Gastric Cancer Patients with Tumors Greater Than 5 cm in Size

Furthermore, we used the Cox regression model to analyze these risk factors in order to identify the independent risk factors for gastric cancer patients. Multivariate analysis revealed that Borrmann type, postoperative chemotherapy, and pathological T stage were independent prognostic factors for these patients (Table 6).

## 4. Discussion

Pathological stage can be used for gastric cancer patients to predict the risk of recurrence and prognosis. Stage I gastric cancer patients have a very low risk of recurrence [11] and are thus not indicated for postoperative chemotherapy. In contrast, stage IV gastric cancer patients can only accept palliative therapy, surgery, chemotherapy, and other treatments [12]. Until now, there has been great variability among the outcomes of patients with stage II/III GC; some patients are prone to suffer from locoregional or distant recurrence even after complete curative resection, whereas others achieve long-term survival [13]. Particularly for stage II gastric cancer patients, the controversy regarding the use of adjuvant chemotherapy following D2 gastrectomy persisted until the completion of the ACTS-GC and CLASSIC trials. The five-year outcomes of the ACTS-GC trial (S-1 versus surgery only) and the CLASSIC trial both indicated that stage II gastric cancer patients can benefit from postoperative chemotherapy [14, 15]. However, in these two clinical trials, the stage II gastric cancer patients included the T2N1M0 and T1N2M0 groups. Moreover, in the CLASSIC trial, the hazard ratio for adjuvant chemotherapy for N0 patients was 0.79 (CI: 0.39–1.60); thus, adjuvant chemotherapy was not advantageous in terms of prognostic improvement. Therefore, whether adjuvant chemotherapy is beneficial for lymph node-negative stage II gastric cancer patients remains unknown.

Because of the controversy regarding the role of postoperative chemotherapy in stage II gastric cancer patients, at our institution, we allowed patients and their relatives to decide whether the patients would receive postoperative chemotherapy. Some patients refused postoperative chemotherapy because of the fear of chemotherapy-related adverse events, and others refused for economic reasons.

In the present study, we demonstrated that adjuvant chemotherapy does not benefit the survival of stage II gastric cancer patients without lymph node metastasis. The median survivals of the patients who did and did not receive adjuvant chemotherapy were 58.0 months and 56.1 months, respectively.

Precision therapy is thought to be the direction of future treatment strategies. Before molecular pathological techniques can be widely used to treat gastric cancer, it is important to determine how stage II gastric cancer patients can be properly selected to receive adjuvant chemotherapy to improve survival.

Although tumor size is not included in the current TNM staging system of the 7th AJCC, this factor still plays an important role in the prediction of the prognoses of gastric cancers due to the ease of its measurement. In Adachi's report, tumor size was strongly correlated with tumor progression parameters, such as the depth of invasion, the degree of lymph node metastasis, and the stage of the disease [16]. Wang et al. suggested that tumor size can efficiently and reliably reflect lymph node status [17]. In the present trial, we found that tumor size was an independent prognostic factor for our group of T_3-4a_N0M0 gastric cancer patients. Moreover, among these T_3-4a_N0M0 gastric cancer patients with tumors greater than 5 cm, adjuvant chemotherapy was an independent prognostic factor. This finding indicates that adjuvant chemotherapy can benefit gastric cancer patients with tumors greater than 5 cm. In our study, we found that, among gastric cancer patients with tumor sizes larger than 5 cm, postoperative chemotherapy improved the prognosis. We therefore propose that postoperative chemotherapy should be performed in this group of patients.

The accurate cancer staging of each patient in clinical practice is crucial for helping clinicians select treatment plans. Although our sample was small, our results imply that tumor size may be useful for guiding adjuvant treatments for T_3-4a_N0M0 gastric cancer patients. However, this study was a retrospective study and thus has limitations, such as confounding factors. Additional experiments and clinical trials are necessary to validate tumor size as a critical factor in determining whether adjuvant chemotherapy should be utilized for T_3-4a_N0M0 patients following D2 gastrectomy.

## Figures and Tables

**Table 1:** Clinical pathological data of the gastric cancer patients.

Clinical pathological data		Small gastric cancer patient group (*n* = 148 cases)		Large gastric cancer patient group (*n* = 121 cases)		*p* value
		Cases	%	Cases	%	
Age (years)	Median	58		62		
	Range	23–79		41–83		
Sex	Male	108	73.0	78	64.5	0.146
	Female	40	27.0	43	35.5	
Tumor location	Gastric cardia	55	37.2	75	62.0	<0.001
	Middle	21	14.2	14	11.6	
	Antrum	66	44.6	21	17.4	
	Total stomach	6	4.1	11	9.1	
CEA level	<5 *μ*g/ml	135	93.1	93	76.9	<0.001
	≥5 *μ*g/ml	10	6.9	28	23.1	
Borrmann type	I	2	1.4	2	1.7	0.145
	II	69	46.6	50	41.3	
	III	77	52.0	65	43.8	
	IV	0	0	4	6.2	
Histological grade	High differentiation	1	0.7	0	0	0.103
	Median differentiation	37	25.0	46	38.0	
	Low differentiation	87	58.8	57	47.1	
	Poor differentiation^∗^	23	15.5	18	14.9	
T staging^∗∗^	T3	130	87.8	97	80.2	0.093
	T4a	18	12.2	24	19.8	
LN harvested	15–29	121	81.8	106	87.6	0.237
	≥30	27	18.2	15	12.4	
Postoperative chemotherapy	Without	56	37.8	33	27.3	0.070
	With	92	62.2	88	72.7	

^∗^Poorly differentiated cells: signet ring cell carcinoma, mucinous adenocarcinoma, undifferentiated carcinoma, etc. ^∗∗^The T and N staging for this group of patients is according to the AJCC 7th TNM staging system for gastric cancer.

**Table 2:** Univariate analysis of the overall survival in this group of gastric cancer patients.

Variables	*n*	Mean survival (months)	*p* value
*Postoperative chemotherapy*			0.543
With	180	58.01	
Without	89	56.08	

*Tumor size*			<0.001
<5 cm	148	63.25	
≥5 cm	121	47.95	

*Tumor location*			0.025
Upper	130	60.01	
Middle	35	46.20	
Lower	87	58.40	
Total	17	48.17	

*Serum CEA level (ng/ml)*			0.529
Normal	228	57.36	
Elevated	38	56.55	

*Borrmann type*			0.119
I	4	68.00	
II	119	59.58	
III	142	54.85	
IV	4	26.75	

*Histological grade*			0.300
High differentiation	1	72.00	
Median differentiation	83	61.71	
Low differentiation	144	61.05	
Poor differentiation	41	46.85	

*T staging*			<0.001
T3	227	59.61	
T4a	42	45.89	

*LN harvested*			0.160
15–29	227	58.31	
≥30	42	51.26	

**Table 3:** Multivariate analyses of overall survival in gastric cancer patients (Cox's regression model).

Variable	HR	95% CI	*p* value
*OS in gastric cancer patients*			
Tumor size	2.780	1.894–4.081	<0.001
CEA level	0.936	0.510–1.717	0.831
Tumor location	1.221	1.023–1.458	0.027
Pathological T staging	2.101	1.342–3.289	0.001

OS, overall survival; HR, hazard ratio; CI, confidence interval.

**Table 4:** Clinical pathological data of the gastric cancer patients whose tumor size is larger than 5 cm.

Clinical pathological data		Without postoperative chemotherapy group (*n* = 33 cases)		With postoperative chemotherapy group (*n* = 88 cases)		*p* value
		Cases	%	Cases	%	
Age (years)	Median	58		62		
	Range	23–79		41–83		
Sex	Male	20	60.6	58	65.9	0.368
	Female	13	39.4	30	34.1	
Tumor location	Gastric cardia	20	60.6	55	62.5	0.639
	Middle	5	15.2	9	10.2	
	Antrum	4	12.1	17	19.3	
	Total stomach	4	12.1	7	8.0	
CEA level	<5 *μ*g/ml	27	81.8	66	75.0	0.296
	≥5 *μ*g/ml	6	18.2	22	25.0	
Borrmann type	I	1	3.0	1	1.1	0.819
	II	12	36.4	38	43.2	
	III	19	57.6	46	52.3	
	IV	1	3.0	3	3.4	
Histological grade	High differentiation	0	0.0	0	0	0.077
	Median differentiation	8	24.2	38	43.2	
	Low differentiation	21	63.6	36	40.9	
	Poor differentiation^∗^	4	12.1	14	15.9	
T staging^∗∗^	T3	25	75.8	72	81.8	0.307
	T4a	8	24.2	16	18.2	
LN harvested	15–29	32	97.0	74	84.1	0.045
	≥30	1	3.0	14	15.9	

^∗^Poorly differentiated cells: signet ring cell carcinoma, mucinous adenocarcinoma, undifferentiated carcinoma, etc. ^∗∗^The T and N staging for this group of patients is according to the AJCC 7th TNM staging system for gastric cancer.

**Table 5:** Univariate analysis of the overall survival in this group of gastric cancer patients.

Variables	*n*	Mean survival (months)	*p* value
*Postoperative chemotherapy*			0.003
With	88	51.23	
Without	33	38.93	

*Tumor location*			0.007
Upper	75	51.68	
Middle	14	34.24	
Lower	21	47.61	
Total	11	36.12	

*Serum CEA level (ng/ml)*			0.105
Normal	93	46.19	
Elevated	28	45.55	

*Borrmann type*			0.039
I	2	66.48	
II	50	53.52	
III	65	43.49	
IV	4	26.75	

*Histological grade*			0.217
High differentiation	0	—	
Median differentiation	46	53.26	
Low differentiation	57	43.27	
Poor differentiation	18	44.77	

*T staging*			<0.001
T3	97	53.39	
T4a	24	26.74	

*LN harvested*			0.479
15–29	106	47.49	
≥30	15	49.29	

**Table 6:** Multivariate analyses of overall survival in gastric cancer patients whose tumor size was larger than 5 cm (Cox's regression model).

Variable	HR	95% CI	*p* value
*OS in gastric cancer patients whose tumor size was larger than 5 cm*			
Borrmann type	1.644	1.039–2.600	0.034
Tumor location	1.116	0.858–1.451	0.414
Pathological T staging	4.761	2.836–9.487	<0.001
Postoperative chemotherapy	0.489	0.281–0.851	0.011

OS, overall survival; HR, hazard ratio; CI, confidence interval.
